# Effects of Tai Chi on executive function, single-leg dynamic balance, and brain functional connectivity in older adults

**DOI:** 10.1038/s41598-025-93321-w

**Published:** 2025-04-07

**Authors:** Xiangyuan Chen, Huifeng Han, Tao Jiang, Guoliang Cai

**Affiliations:** Department of Sports and Human Sciences, Harbin Sport University, Harbin, 150008 PR China

**Keywords:** Dynamic balance ability, Brain functional connectivity, Lateralization, Y balance tests, Brisk walking exercise, Anatomy, Musculoskeletal system, Nervous system, Psychology, Human behaviour, Geriatrics, Public health, Therapeutics

## Abstract

Although previous studies have confirmed the beneficial effects of Tai Chi Chuan (TCC) on improving balance ability and cognitive function in older adults, the synergistic regulatory mechanisms of TCC on bilateral lower limb muscle activation symmetry, brain functional connectivity(FC) strength, and dynamic balance ability remain unclear. This study aims to investigate the effects of a 9-week TCC training program on the executive function (EF), bilateral lower limb muscle activation symmetry, single-leg dynamic balance ability, and brain FC strength in elderly individuals. It provides valuable new insights into a field that has not been extensively explored before. After 9-week training intervention, (1) The TCC group showed significant improvements in the Y-balance Test (YBTs), with enhanced symmetry in single-leg dynamic balance between the bilateral lower limbs and a significant reduction in the laterality of lower limb muscle activation. (2) TCC training strengthened the FC of related brain regions during YBTs. When performing YBTs with the left and right legs separately, the laterality of the average brain FC strength between the two tasks was significantly reduced. (3) During EF tasks, the reaction time was significantly reduced, and the concentration of oxygenated hemoglobin in the prefrontal cortex increased. Studies have shown that TCC has significantly superior intervention effects compared to brisk walking in improving neuromuscular function in older adults. Through its multisensory integration training model, TCC simultaneously enhances neuromuscular coordination and brain network collaboration efficiency, promoting the adaptive reorganization of dynamic balance control.

**Supplementary Information:**

The online version contains supplementary material available at 10.1038/s41598-025-93321-w.

## Introduction

With the intensification of global population aging, falls have become a significant public health challenge affecting the health and quality of life of the elderly. They are a major risk factor for soft tissue injuries, fractures, traumatic brain injuries, loss of independence, and even mortality among older adults^1^. Approximately 50% of falls occur during single-leg support phases, such as when stepping over obstacles or climbing stairs^2^. During the aging process, in addition to factors such as loss of muscle strength and declining vision, the degeneration of nervous system function and the reduction in bilateral lower limb muscle strength symmetry also increase the risk of falls among the elderly^3,4^. Furthermore, prolonged sedentary behavior(SB) may exacerbate the decline in nervous system function and lower limb muscle strength during aging^5^, thereby further increasing the incidence of falls among older adults^6^.

Long-term SB and aging, among other factors, can lead to volume loss in white matter (WM) and gray matter (GM), as well as an increase in white matter lesions, resulting in brain atrophy. This is primarily characterized by a reduction in neuronal projections, decreased efficiency of brain functional connectivity(FC), weakened motor control, and impaired cognitive function^7,8^. Previous studies have found that poor brain FC efficiency and cognitive function can impair the ability of the elderly to process motor information in the nervous system, particularly in fine motor skills and balance control, making older adults more prone to postural instability, delayed reactions, and motor incoordination^9^. In addition to the degeneration of nervous system function, the weakening and asymmetry of bilateral lower limb muscle strength can also contribute to reduced balance control in the elderly, often manifesting as abnormal gait patterns (such as unsteady gait, uneven stride length, or excessively fast or slow steps)^10^. Therefore, exploring effective interventions to improve the symmetry of lower limb muscle strength and enhance nervous system function is of significant importance for improving motor abilities, reducing fall risks, and enhancing the overall quality of life in the elderly population.

Neuroplasticity theory posits that the structure and function of the brain can undergo self-adjustment and remodeling in response to regular changes in internal and external environments^11^. Previous studies have found that exercise can elevate levels of neurotrophic factors in the human body, promote synaptic plasticity, and enhance communication efficiency between neurons^12–14^ For example, compared to sedentary older adults, those who engage in regular physical activity exhibit enhanced neuroplasticity, manifested as increased GM and WM volumes in the brain and heightened activation levels in cortical regions during tasks^15^. Based on the theory of angiogenesis, researchers have discovered that exercises with complex movement structures can effectively promote the generation of microvasculature in the cerebral cortex, thereby improving cerebral blood supply to support neuronal growth, repair, and functional maintenance^16^. Tai Chi Chuan(TCC), a mind-body exercise that integrates aerobic activity with meditation training, involves a series of complex movement structures. It emphasizes rhythmic rotation of the torso, precise control of body weight shifts, and the relative positioning of limbs^17,18^. TCC can effectively improve the input-output relationship of somatic receptors, enhance the efficiency of signal transmission, and optimize the structure and function of neural networks^19^. Long-term practice of TCC not only increases lower limb muscle strength and dynamic balance but also boosts brain volume and cortical thickness^20^. The meditation component of TCC can also elevate dopamine levels and synaptic plasticity in cortical regions such as the striatum, hippocampus, and prefrontal cortex^21^, positively impacting overall cognitive function, executive function(EF), attention, and memory in practitioners^22,23^.

Given that neuroplasticity can enhance behavioral performance, it is reasonable to hypothesize that behavioral changes associated with TCC practice should be detectable in corresponding brain regions. Non-invasive neuroimaging techniques provide researchers with tools to identify the neural correlates of brain changes. Compared to functional magnetic resonance imaging (fMRI), functional near-infrared spectroscopy(fNIRS) offers superior portability, real-time monitoring, and flexibility during movement^24^. This allows for the detection of oxygenated hemoglobin(HbO) concentrations in cortical regions during physical activities, enabling the inference of exercise-induced brain activation levels and FC strength.

TCC, as a comprehensive exercise intervention, holds significant potential for improving dynamic balance and enhancing neurological function. However, existing neuroimaging studies on TCC have primarily focused on static FC, with limited exploration of dynamic FC, thus failing to provide a complete picture of brain plasticity changes. Whether TCC exerts superior effects on brain plasticity compared to general aerobic exercise remains unclear and warrants further investigation. Therefore, the primary aim of this study is to investigate whether a 9-week TCC training program can induce changes in brain FC efficiency, EF, symmetry of single-leg dynamic balance between bilateral lower limbs, and symmetry of muscle activation in older adults, and whether these changes are superior to those induced by aerobic exercise. We hypothesize that, compared to brisk walking(BW), a 9-week TCC intervention will significantly improve EF, single-leg dynamic balance, symmetry of muscle activation in bilateral lower limbs, connectivity strength between regions of interest, and overall symmetry of average brain FC in older adults.

## Materials and methods

### Participants

This study is a randomized controlled trial (RCT) that involved 90 SB elderly individuals (58 males and 32 females) who met the inclusion criteria. Participants were stratified and randomly assigned to one of three groups based on the participant characteristic data, using computer-generated randomization. The three groups were the TCC group (*n* = 30, 19 males and 11 females), the BW group (*n* = 30, 20 males and 10 females), and the control group (*n* = 30, 19 males and 11 females). The inclusion criteria for participants were as follows: (1) Individuals aged between 60 and 70 years with a Montreal Cognitive Assessment (MoCA) score of 26 or higher; (2) For the past 6 months, participants had not engaged in TCC, BW, or any other irregular physical exercise, except for routine daily activities, and had a daily SB time of ≥ 6 h; (3) Right-handed as assessed by the Edinburgh Handedness Inventory, with no vestibular disorders, color blindness, or visual impairments; (4) Blood pressure has been well-controlled over the past two years, with no significant cardiovascular, pulmonary, or metabolic diseases. (5) Participants were excluded if they had long-term medication usage, cognitive disorders, or severe musculoskeletal diseases; (6) In the YBTs, the lateral muscle activation asymmetry index of the left and right quadriceps and hamstrings was ≥ 15%^25^. When the muscle activation asymmetry between the two lower limbs exceeds 15%, it can negatively impact physical function^26,27^. The asymmetry index was calculated using the following formula: Asymmetry Index = |(Left - Right)| / (Left + Right). This study was approved by the Human Experiment Ethics Committee of Harbin Sport University (Approval No: 2024014). All Participants signed written informed consent in accordance with the Declaration of Helsinki. Table 1 presents the characteristics of the participants.

**Table 1:** Characteristics of participants (Mean ± SD).

	Control group	BW group	TCC group	*P*-value
Age(years)	64.13 ± 4.06	65.36 ± 3.02	64.3 ± 4.2	
Education years(years)	8.77 ± 1.45	8.57 ± 1.41	8.8 ± 1.24	0.776
MoCA	27.93 ± 1.17	27.77 ± 1.17	28.17 ± 1.09	0.399
ST(hour/day)	7.78 ± 0.62	7.61 ± 0.8	7.66 ± 0.66	0.612
PA(MET-min/week)	589.67 ± 28.46	588.33 ± 41.94	603.67 ± 37.28	0.2

*ST* sedentary time,* PA * physical activity.

### Experimental procedure

One week prior to the official start of the experiment, participants were asked to wear loose and comfortable clothing and come to the laboratory in the morning to familiarize themselves with the testing procedures and undergo baseline assessments (pre-test). This study adopts a double-blind design. Before testing, each participant is assigned and recorded a random number to ensure that both participants and evaluators are unaware of the specific group allocation. This approach prevents group information from influencing the testing process, thereby ensuring the fairness and objectivity of the study results. During the baseline assessment, participants’ years of education were recorded, and the MoCA was used to screen for cognitive function to ensure all participants had similar cognitive levels. Additionally, the International physical activity questionnaire-short form (IPAQ-SF) was used to assess participants’ sedentary time and physical activity levels. Repeated measurements (post-test) were conducted within one week after the 9th week, during the morning. Before and after the training, participants’ EF was measured, and fNIRS monitoring was conducted during the EF tests. The YBTs was primarily used to assess single-leg balance, with simultaneous fNIRS and surface electromyography (sEMG) monitoring during the YBTs. The test for each project was repeated three times, and the maximum value for each participant was recorded. Prior to each test, participants were instructed to sleep before 23:00 the night before to ensure adequate rest. They were required to fast for 4 h before the test, and to avoid consuming tea or caffeine-containing foods for the previous 6 h. To prevent measurement errors, all tests were performed by the same evaluator. The intervention was carried out at Harbin Children’s Park. During the study, participants in all three groups were instructed to avoid engaging in any regular physical exercise and to maintain their usual dietary habits. Additionally, each participant was required to attend at least 36 sessions (80% attendance) of training.

The training protocol for the TCC group and the BW group were designed to be conducted five times a week for a duration of nine weeks. This training period was determined based on findings from previous studies, which indicated that this timeframe is sufficient to induce significant improvements in both physical and cognitive functions among the elderly population^28–30^. In contrast, the control group was instructed to maintain their original sedentary lifestyle. During the intervention period, the training intensity for the TCC and BW groups was set at 60–70% of their maximum heart rate, which was calculated using the formula 220 minus their age. This intensity corresponded to approximately 13 points on the Borg Rating of Perceived Exertion scale (ranging from 6 to 20). Participants in the TCC and BW groups underwent five training sessions in the first week (adaptation training). The 24-form simplified TCC was chosen for its ease of learning and its widely recognized effectiveness in enhancing both physical and cognitive health. Therefore, the TCC group practiced the 24-form simplified TCC under the guidance of a professional TCC instructor. Each training session consisted of a 5-minute warm-up, 30 min of learning new movements, 10 min of reviewing previously learned movements, and 5 min of relaxation exercises. In the BW group, participants were instructed to walk within the training intensity range at a speed of 1.24–1.38 m/s^31^. Each training session included 5 min of warm-up, with walking time gradually increasing from 20 min to 40 min (To ensure that participants could maintain a 40-minute training duration in the subsequent 8 weeks of training), followed by 5 min of relaxation. If a participant reached the training intensity but did not achieve the target speed range, the speed requirement could be lowered slightly. Conversely, if the participant exceeded the speed range without reaching the desired intensity, walking speed could be increased within a safe range. The walking speed and pacing were adjusted by a professional coach to ensure the BW participants stayed within the appropriate range. In the subsequent 8 weeks of formal training, the participants in both the TCC and BW groups continued to train under the guidance of professional instructors. Each session began with 5 min of warm-up, followed by 40 min of either TCC or BW, and ended with 5 min of relaxation exercises. The intensity of each intervention session was controlled by the coach, who adjusted the walking speed and pacing for the BW group and regulated the intensity for the TCC group using steps, body movements, and postures to ensure that participants achieved the required training intensity.

### Measurement

#### EF tests

The EF refers to the psychological process in which the body controls thoughts and behaviors through consciousness, and is an important component of cognitive function. For testing the EF, we mainly use the Stroop task and the 2-back task. These tasks are programmed in E-prime 3.0 software, which records reaction times(RTs) and Correct rates(CR) to assess the participant’s working memory and inhibitory control abilities. Before the EF tests begin, participants from all three groups are required to practice these two tasks continuously until they achieve an accuracy rate of over 85% before starting the formal experiment. The test includes four blocks, each containing 12 trials, with a 30-second interval between blocks. The letter presentation time is 1.5 s, with a stimulus interval of 1 s, and the total test duration is 3 min and 30 s. ( For testing details, please refer to Appendix A.1 )

#### fNIRS data

The fNIRS data were recorded using a multi-channel portable Nirsmart system (NirScan-3000 C, Huichuang Medical Equipment Co., Ltd., Danyang, China). This system utilizes two wavelengths (730 nm and 850 nm), with a sampling frequency of 11 Hz. The experimental cap is designed based on the 10/20 international standard electrode placement system, and is available in three sizes (large, medium, and small) to accommodate participant’s head circumferences. The cap is placed on the participant’s head, with the Cz location as the reference point, followed by visual alignment checks in the sagittal plane. Each participant’s probe set consists of 16 detectors (sensors) and 16 emitters (light sources), forming a total of 39 channels. The distance between the detectors and emitters is 30 mm^32^. The system effectively covers and collects changes in the oxyhemoglobin signal in the prefrontal cortex(PFC) and motor cortex regions, reflecting both spontaneous and task-related brain activity. This setup is suitable for analyzing functional brain FC during motor tasks. Before the experiment begins, it is essential to ensure that the signals from all channels within the region of interest meet the required quality standards. If any channel fails to meet these standards, the experimental testing cannot proceed. Prior to the YBTs (Y Balance Tests), participants are required to stand quietly with their eyes closed in a quiet testing room, and a 5-second baseline is recorded to obtain the relative changes in HbO (oxygenated hemoglobin) concentration. The brain regions measured were divided into six ROIs: the Premotor Cortex (PMC), Supplementary Motor Area (SMA), Primary Motor Cortex (M1), Primary Sensory Cortex (S1), Frontal Pole Area (FPA), and Dorsolateral Prefrontal Cortex (DLPFC). PMC and SMA were monitored together due to their close proximity. The channels for PMC and SMA are (CH1, CH15, CH18, CH28, CH29, CH31, CH32, CH35, CH38, CH39). The channels for M1 are (CH17, CH33, CH30, CH34, CH36, CH37). The channels for S1 are (CH2, CH13, CH14, CH16). The channels for DLPFC are (CH4, CH12, CH19, CH20, CH25, CH27). The channels for FPA are (CH7, CH10, CH22, CH23, CH24). (The data analysis methods for fNIRS are detailed in Appendix A.2)

**Fig. 1:**
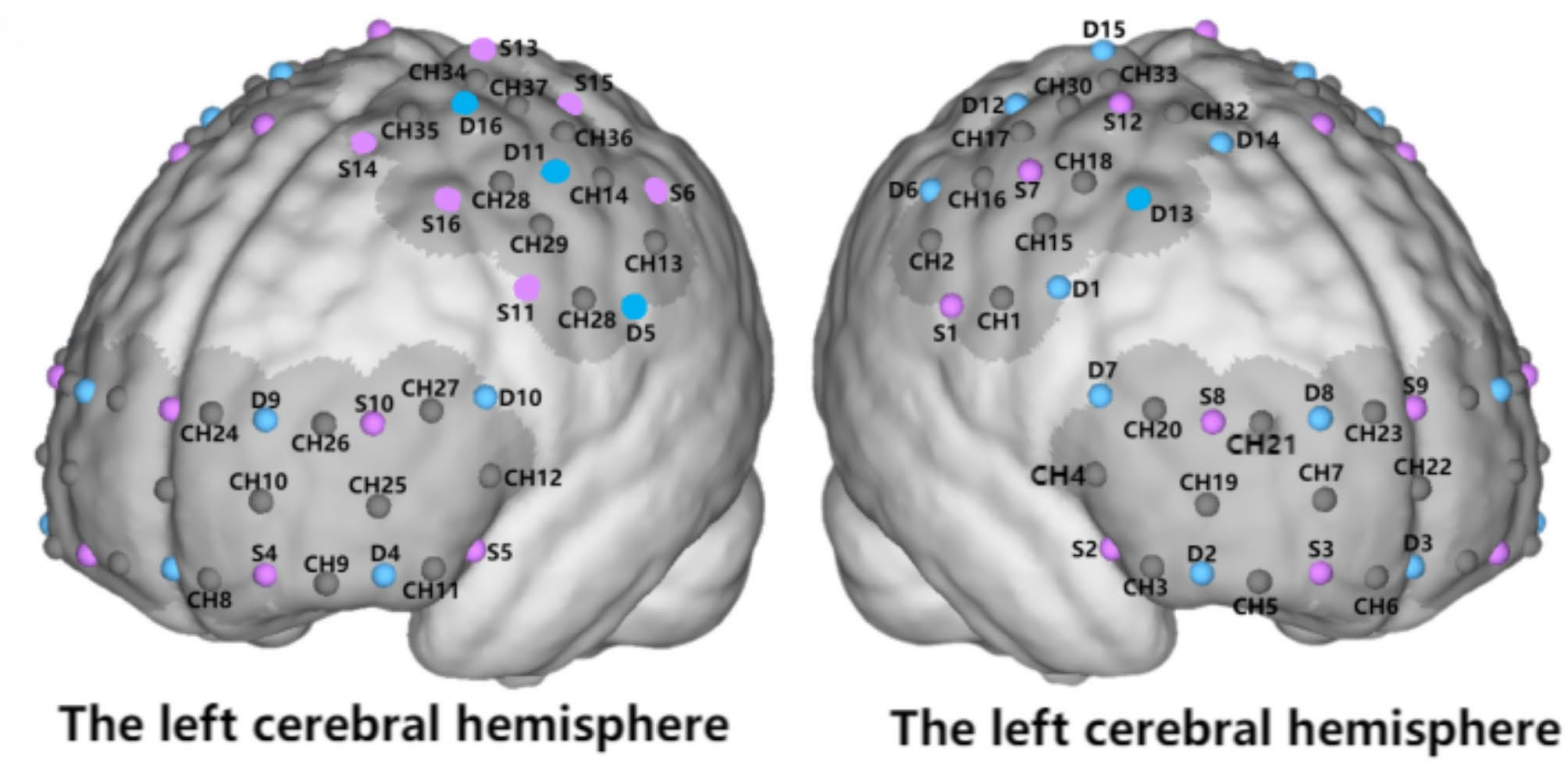
The near-infrared spectroscopy probe is configured with detectors (D), sources (S), and channels (CH).

#### YBTs

The YBTs is a functional assessment method used to evaluate dynamic single-leg balance capabilities. The YBTs single-leg dynamic balance test reflects the overall level of the body, including neuromuscular coordination, muscle strength, body perception, central nervous system function, trunk core muscle stability, and the coordination between both sides of the body^33,34^. Research has found that such challenging postural tasks can amplify asymmetries in the body, while static single-leg standing tests lack sufficient challenge^35,36^. After participants became familiar with the testing procedures, they were required to perform the test three times in each direction. The maximum reach distance for each participant was recorded, and the composite score(CS) was calculated. To ensure safety during the test, two safety officers are present to prevent falls. (For the specific testing protocol and the formula for calculating the CS, please refer to Appendix A.3)

#### Wireless sEMG tests

The sEMG signals are collected using the YW-Wireless system (Wuhan Zhiti Technology Co., Ltd.). Before placing the electrodes, alcohol is used to clean the skin to remove grease, reduce skin impedance, and improve conductivity. Surface electrodes (50 mm diameter Ag-AgCl electrodes) are affixed to the surface of the skin over the muscles, with an inter-electrode distance of 2 cm. To ensure high-quality sEMG signals, the electrode positions are determined according to the guidelines outlined in the SENIAM protocol^37^. The muscles selected for sEMG collection are the vastus lateralis(VL) and biceps femoris(BF). These muscles play critical roles in lower limb movements, exhibiting strong electrical activity that is easily captured by surface electrodes. Additionally, their anatomical positions make them ideal for sEMG collection. The sEMG signals are subjected to band-pass filtering (20–450 Hz, 4th-order Butterworth filter) and then integrated electromyographic (iEMG) values are calculated based on the YBTs testing time for each participant. The increase in iEMG is positively correlated with muscle contraction intensity, and the iEMG values are used to reflect changes in muscle strength during the YBTs process in older adults. (The calculation steps for Normalized iEMG and CCI can be found in Appendix A.4.)

### Statistical analysis

Statistical analysis was performed using SPSS 26.0 software, and data visualization was conducted using GraphPad Prism 9. Normally distributed measurement data were described as mean ± standard deviation (Mean ± SD). One-way analysis of variance (ANOVA) was used to confirm that there were no significant differences in the baseline characteristics of participants across groups. A two-way ANOVA was employed to compare reaction times and accuracy rates in executive function tasks, oxygenated hemoglobin concentrations in the prefrontal cortex, CSs of YBTs, laterality indices of functional connectivity strength between regions of interest during YBTs, overall average laterality indices of functional connectivity strength, and laterality indices of integrated electromyographic values of the biceps femoris and vastus lateralis muscles during YBTs. Post hoc pairwise comparisons were performed using the Bonferroni method. A two-tailed test was used, and statistical significance was considered at *P* ≤ 0.05.

## Results

### YBTs results

According to the results of the two-way ANOVA presented in Table 2, during the YBTs task with the right leg stance, there was a significant difference in the CS (F_group_=5.118, *P* = 0.007) among the three groups(*P* < 0.05). Post-hoc pairwise comparisons using the Bonferroni method revealed that the post-test CS in the TCC group (0.006) was significantly higher than that in the control group, with statistical significance. However, no significant differences were observed in the CS between the pre-test and post-test across the three groups (*P* > 0.05). In the YBTs task with the left leg stance, the CS (F_group_=6.588, *P* = 0.002) among the three groups also showed a significant difference(*P* < 0.05). Post-hoc pairwise comparisons using the Bonferroni method indicated that the post-test CS in both the TCC group(0.003) and the BW group(0.013) was significantly better than that in the control group, with statistical significance. Additionally, significant differences were observed in the CS between the pre-test and post-test across the three groups (F_time_=9.548, *P* = 0.002), with the post-test CS in the TCC group and the BW group showing significant improvement. The interaction effect between group and time (F_group*time_=4.706, *P* = 0.01) also reached a significant level (*P* < 0.05).

### Results of the wireless sEMG

According to the results of the two-way ANOVA presented in Table 2, during the YBTs task with the right leg as the supporting leg, the normalized iEMG values of the VL in the right supporting leg showed significant differences among the three groups (F_group_ = 3.891, *P* = 0.022), post-hoc analysis using the Bonferroni method revealed that the post-test normalized iEMG values of the right supporting leg VL in the TCC group were significantly higher than those in the control group (*P* = 0.049), with statistical significance(*P* < 0.05). However, no significant differences were observed in the normalized iEMG values of the right supporting leg VL among the three groups between the pre-test and post-test (*P* > 0.05). For the normalized iEMG values of the BF in the right supporting leg during the YBTs task, significant differences were found among the three groups (F_group_ = 8.004, *P* = 0.001), and the interaction effect between group and time (F_group*time_ = 3.373, *P* = 0.037) was also significant (*P* < 0.05). Post-hoc analysis using the Bonferroni method indicated that the post-test normalized iEMG values of the right supporting leg BF in both the TCC group (*P* = 0.002) and the BW group (*P* = 0.003) were significantly higher than those in the control group, with statistical significance. However, no significant differences were observed in the normalized iEMG values of the right supporting leg BF among the three groups between the pre-test and post-test (*P* > 0.05).

During the YBTs task with the left leg as the supporting leg, the normalized iEMG values of the VL in the left supporting leg showed significant differences among the three groups (F_group_ = 4.815, *P* = 0.009), post-hoc analysis using the Bonferroni method revealed that the post-test normalized iEMG values of the left supporting leg VL in the TCC group were significantly higher than those in the control group (*P* = 0.008), with statistical significance(*P* < 0.05). Additionally, the interaction effect between group and time (F_group*time_ = 7.42, *P* = 0.001) and the differences between the pre-test and post-test (F_time_ = 4.729, *P* = 0.031) in the normalized iEMG values of the left supporting leg VL were also significant, with the post-test values in the TCC group showing a significant increase (*P* < 0.05). However, no significant differences were observed in the normalized iEMG values of the left supporting leg BF among the three groups or between the pre-test and post-test (*P* > 0.05).

### Results of the CCI

According to the results of the two-way ANOVA presented in Table 2, during the YBTs task with the right leg as the supporting leg, there was a significant difference in the CCI of the right supporting leg among the three groups (F_group_ = 9.315, *P* = 0.001), and the interaction effect between group and time (F_group*time_ = 6.487, *P* = 0.002) was also significant (*P* < 0.05). Post-hoc pairwise comparisons using the Bonferroni method revealed that the post-test CCI of the right supporting leg in the TCC group was significantly higher than that in the control group (*P* = 0.001), with statistical significance. However, no significant differences were observed in the CCI of the right supporting leg among the three groups during the pre-test and post-test (*P* > 0.05). In the YBTs task with the left leg as the supporting leg, there was a significant difference in the CCI of the left supporting leg among the three groups (F_group_ = 7.483, *P* = 0.001), and the interaction effect between group and time (F_group*time_ = 5.294, *P* = 0.006) was also significant (*P* < 0.05). Post-hoc pairwise comparisons using the Bonferroni method indicated that the post-test CCI of the left supporting leg in the TCC group was significantly higher than that in the control group (*P* = 0.001), with statistical significance (*P* < 0.05). However, no significant differences were observed in the CCI of the right supporting leg among the three groups during the pre-test and post-test (*P* > 0.05).

**Table 2:** Changes in the CS of the YBTs, RTS and CR in EF tests (Mean ± SD).

		Control group		BW group		TCC group		*P*_*group*_	*P*_*time*_
		Pre-test	Post-test	Pre-test	Post-test	Pre-test	Post-test		
CS	Right leg	0.74 ± 0.03	0.734 ± 0.03	0.744 ± 0.03	0.753 ± 0.02	0.743 ± 0.03	0.764 ± 0.04	**0.007**	0.065
	Left leg	0.734 ± 0.03	0.729 ± 0.02	0.737 ± 0.03	0.757 ± 0.02	0.74 ± 0.04	0.763 ± 0.03	**0.002**	**0.002**
iEMG	R-VL	0.608 ± 0.06	0.594 ± 0.06	0.59 ± 0.05	0.611 ± 0.05	0.613 ± 0.06	0.638 ± 0.05	**0.022**	0.192
	R-BF	0.617 ± 0.06	0.605 ± 0.06	0.635 ± 0.04	0.651 ± 0.05	0.626 ± 0.05	0.663 ± 0.05	**0.001**	0.077
	L-VL	0.625 ± 0.05	0.602 ± 0.04	0.615 ± 0.06	0.634 ± 0.06	0.616 ± 0.06	0.679 ± 0.09	**0.009**	**0.031**
	L-BF	0.606 ± 0.06	0.599 ± 0.06	0.608 ± 0.06	0.627 ± 0.06	0.611 ± 0.09	0.632 ± 0.04	0.21	0.237
CCI	Right leg	0.669 ± 0.05	0.635 ± 0.03	0.664 ± 0.04	0.676 ± 0.05	0.677 ± 0.06	0.704 ± 0.06	**0.001**	0.002
	Left leg	0.657 ± 0.05	0.633 ± 0.04	0.659 ± 0.05	0.673 ± 0.05	0.673 ± 0.07	0.7 ± 0.07	**0.001**	**0.006**
Stroop task	RTs (ms)	622.61 ± 72.39	618.75 ± 65.57	626.83 ± 74.89	602.24 ± 67.11	633.98 ± 61.96	583.43 ± 61.96	0.624	**0.01**
	CR (%)	0.917 ± 0.03	0.92 ± 0.03	0.915 ± 0.04	0.917 ± 0.04	0.918 ± 0.03	0.926 ± 0.05	0.674	0.445
2-back task	RTs (ms)	634.06 ± 78.67	640.1 ± 60.93	637.11 ± 71.27	609.1 ± 63.66	641.06 ± 68.62	601.95 ± 60.81	0.384	**0.045**
	CR (%)	0.918 ± 0.04	0.92 ± 0.04	0.924 ± 0.04	0.926 ± 0.05	0.917 ± 0.04	0.923 ± 0.04	0.713	0.608

Bold text indicates significant differences with *P* < 0.05.

### EF test RTS and CR result

According to the results of the two-way ANOVA presented in Table 2, in the Stroop task, the RTs among the three groups did not show significant differences (*P* > 0.05). However, there was a significant difference in RTs between the pre-test and post-test (F_time_=6.862, *P* = 0.01), with the post-test RTs in the TCC group significantly shortened. Additionally, the CR among the three groups did not show significant differences in either the pre-test or post-test and time was also not significant (*P* > 0.05). In the 2-back task, the RTs among the three groups also did not show significant differences (*P* > 0.05). However, there was a significant difference in RTs between the pre-test and post-test (F_time_ = 9.548, *P* = 0.002), with the post-test RTs in both the BW group and the TCC group significantly shortened. The CR among the three groups did not show significant differences in either the pre-test or post-test (*P* > 0.05).

### Results of changes in HbO concentration in the PFC during EF tests

**Fig. 2:**
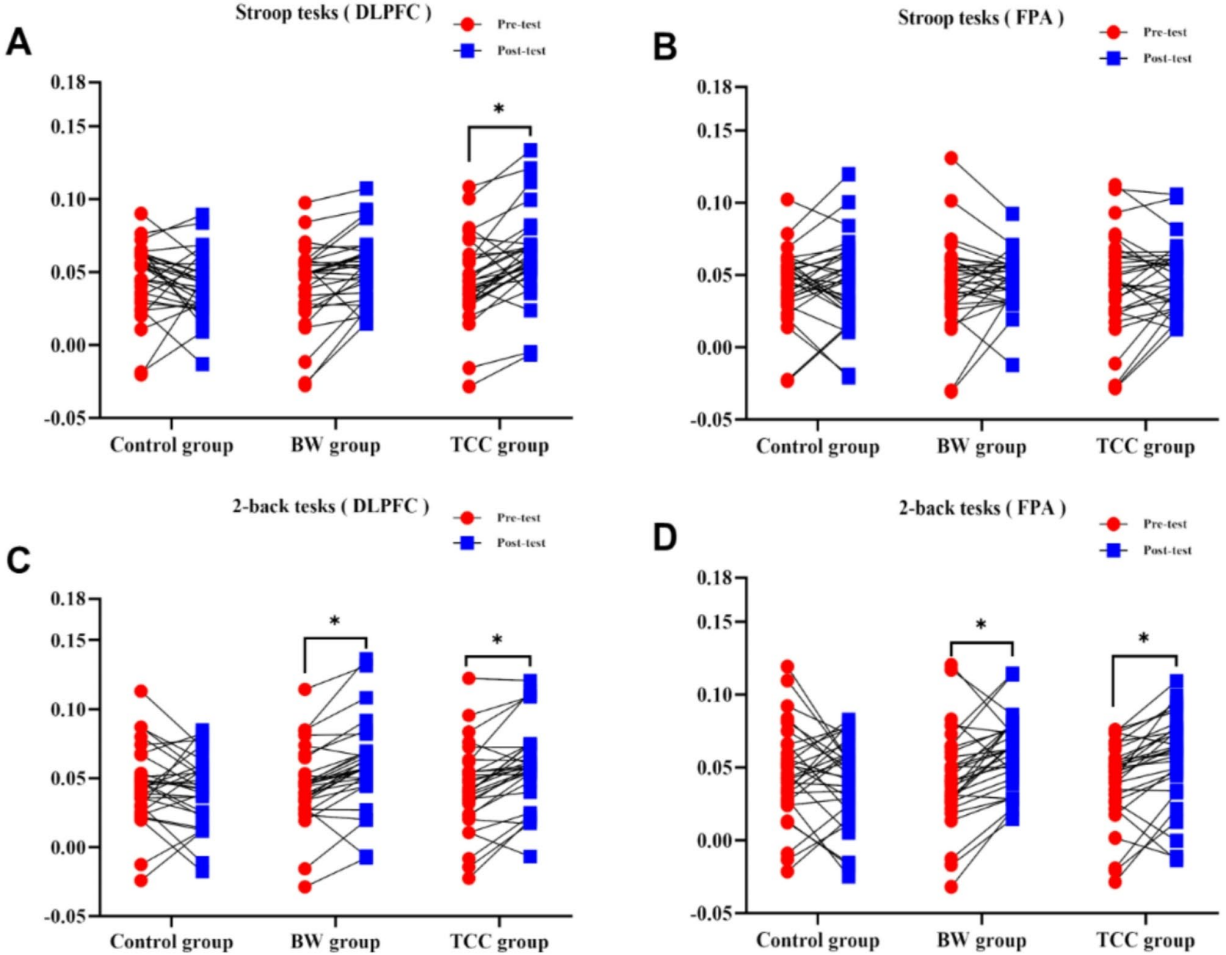
Changes in HbO concentration in the PFC during stroop and 2-back tasks. (**A**): Changes in HbO concentration in the DLPFC during the Stroop task. (**B**): Changes in HbO concentration in the FPA during the Stroop task. (**C**): Changes in HbO concentration in the DLPFC during the 2-back task. (**D**): Changes in HbO concentration in the FPA during the 2-back task. * indicates a significant difference between pre-test and post-test values within the group (* *p* < 0.05).

A two-way ANOVA revealed that in the Stroop task, there were no significant differences in the HbO concentration in the DLPFC brain region among the three groups (*P* > 0.05). However, a comparison between the pre-test and post-test showed a significant difference in the HbO concentration in the DLPFC ( F_time_ = 4.053, *P* = 0.046 )brain region (*P* < 0.05). Specifically, as illustrated in Fig. 2A, the HbO concentration in the DLPFC region of the TCC group significantly increased during the post-test. Additionally, no significant differences were observed in the HbO concentration in the FPA brain region either between groups or between the pre-test and post-test (*P* > 0.05). In the 2-back task, as shown in Fig. 2C and D, no significant differences were observed in the HbO concentration in the DLPFC and FPA brain regions among the three groups (*P* > 0.05). However, a comparison between the pre-test and post-test revealed significant differences in the HbO concentration in both the DLPFC (F_time_ = 5.137, *P* = 0.025) and FPA (F_time_ = 4.446, *P* = 0.036) brain regions (*P* < 0.05). Specifically, the HbO concentration in the DLPFC and FPA regions significantly increased in both the BW group and the TCC group.

### Brain FC results between rois during right leg Y-balance tests

**Fig. 3:**
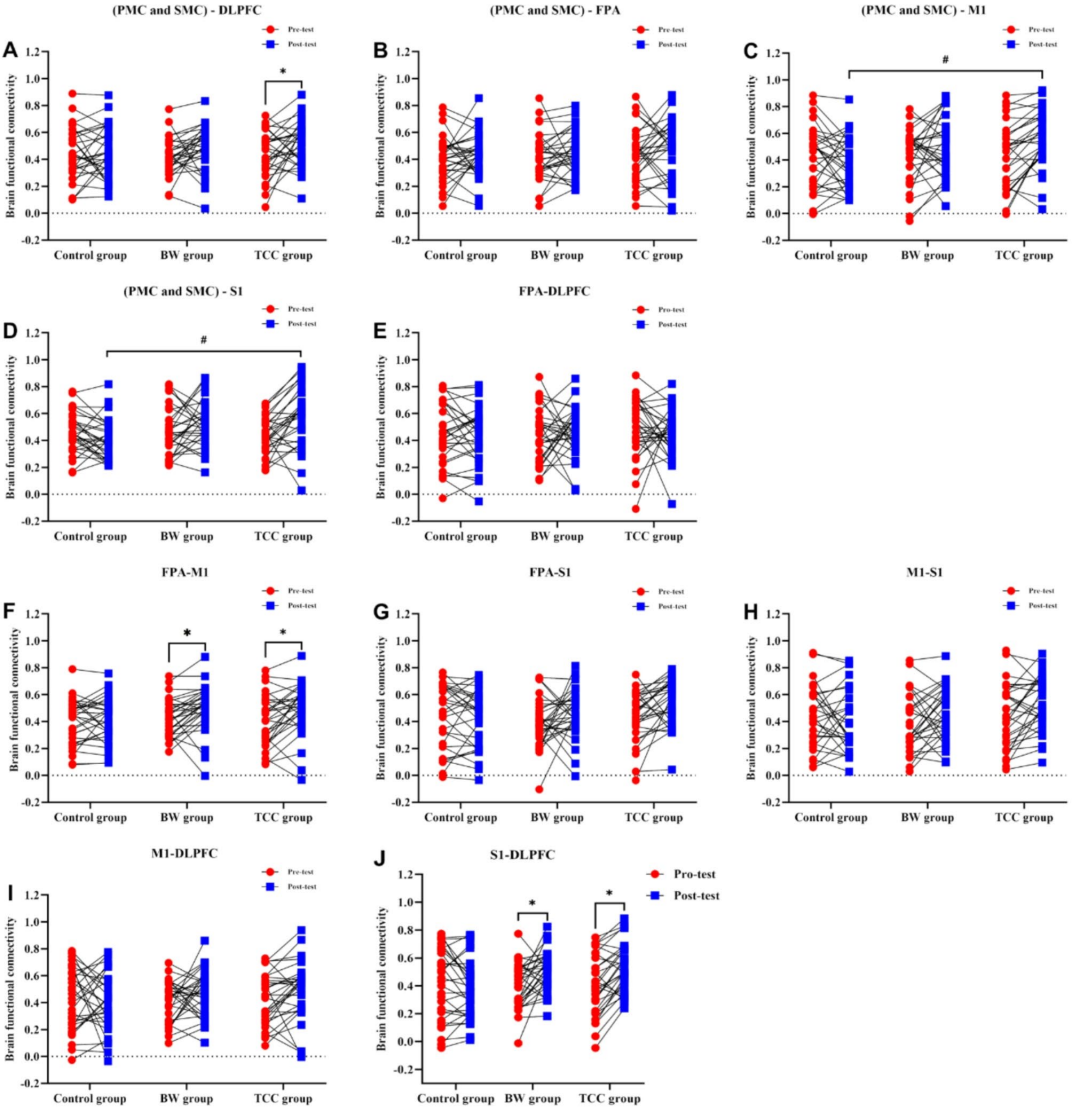
Changes in pre-test and post-test values of brain FC between areas of interest during right Leg YBTs. (**A**): Changes in FC strength between (PMC and SMC)-DLPFC, (**B**): Changes in FC strength between (PMC and SMC)-FPA, (**C**): Changes in FC strength between (PMC and SMC)-M1, (**D**): Changes in FC strength between (PMC and SMC)-S1, (**E**): Changes in FC strength between FPA-DLPFC, (**F**): Changes in FC strength between FPA-M1, (**G**): Changes in FC strength between FPA-S1, (**H**): Changes in FC strength between M1-S1, (**I**): Changes in FC strength between M1-DLPFC, (**J**): Changes in FC strength between S1-DLPFC. * represents significant differences within groups between pre-test and post-test (* *p* < 0.05). # denotes a significant difference between groups (#, *p* < 0.05).

According to the two-way ANOVA results in Fig. 3A, during the YBTs task with right leg support, there were no significant differences in the FC strength between the (PMC and SMC)-DLPFC brain regions among the three groups (*P* > 0.05). However, the comparison between pre-test and post-test (F_time_ = 4.674, *P* = 0.032) revealed a significant difference in the FC strength between the (PMC and SMC)-DLPFC brain regions (*P* < 0.05), with the TCC group showing a significant increase in FC strength during the post-test. Figure 3C shows a significant difference in the FC strength between the (PMC and SMC)-M1 brain regions among the three groups (F_group_ = 3.095, *P* = 0.048). Bonferroni post-hoc comparisons indicated that the post-test FC strength between the (PMC and SMC)-M1 brain regions in the TCC group(0.046) was significantly higher than that in the control group (*P* < 0.05). However, there were no significant differences in the FC strength between the (PMC and SMC)-M1 brain regions in the three groups during the pre-test and post-test (*P* > 0.05). Figure 3D shows a significant difference in the FC strength between the (PMC and SMC)-S1 brain regions among the three groups (F_group_ = 3.251, *P* = 0.041). Bonferroni post-hoc comparisons revealed that the post-test FC strength between the (PMC and SMC)-S1 brain regions in both the TCC group(0.036) was significantly higher than that in the control group. Additionally, the interaction effect between group and time (F_group*time_ = 4.448, *P* = 0.013) was also significant (*P* < 0.05). However, there were no significant differences in the FC strength between the (PMC and SMC)-S1 brain regions in the three groups during the pre-test and post-test (*P* > 0.05). Figure 3F shows no significant differences in the FC strength between the FPA-M1 brain regions among the three groups (*P* > 0.05). However, there was a significant difference in the FC strength between the FPA-M1 brain regions during the pre-test and post-test (F_time_ = 5.332, *P* = 0.022) (*P* < 0.05), with both the BW group and the TCC group showing a significant increase in FC strength during the post-test. In Fig. 3J, there were no significant differences in the FC strength between the S1-DLPFC brain regions among the three groups (*P* > 0.05). However, there was a significant difference in the FC strength between the S1-DLPFC brain regions during the pre-test and post-test (F_time_ = 4.127, *P* = 0.044) (*P* < 0.05), with both the BW group and the TCC group showing a significant increase in FC strength during the post-test. In Fig. 3B, E, G and H, and 3I, FC strength between the (PMC and SMC)-FPA, FPA-DLPFC, FPA-S1, M1-S1, and M1-DLPFC brain regions showed no significant differences among the groups, nor were there significant differences between the pre-test and post-test comparisons (*P* > 0.05).

### Brain FC results between regions of interest during YBTs of the left leg

**Fig. 4:**
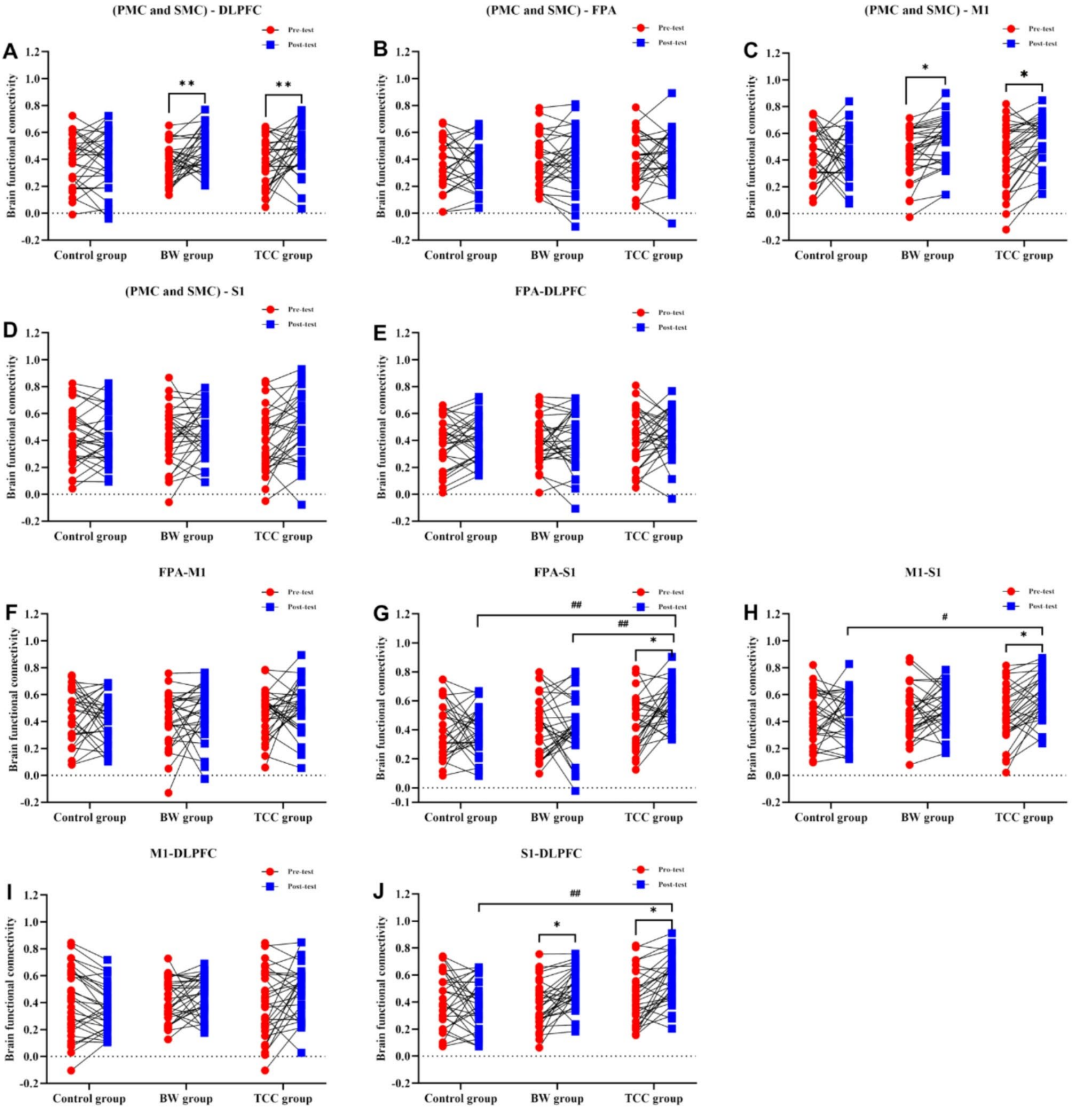
Changes in pre-test and post-test values of brain FC between different regions of interest during YBTs of the left leg. Figure 4. Changes in the pre-test and post-test values of brain FC strength between different regions of interest during YBTs for the left leg. (**A**): Changes in FC strength between (PMC and SMC)-DLPFC, (**B**): Changes in FC strength between (PMC and SMC)-FPA, (**C**): Changes in FC strength between (PMC and SMC)-M1, (**D**): Changes in FC strength between (PMC and SMC)-S1, (**E**): Changes in FC strength between FPA-DLPFC, (**F**): Changes in FC strength between FPA-M1, (**G**): Changes in FC strength between FPA-S1, (**H**): Changes in FC strength between M1-S1, (**I**): Changes in FC strength between M1-DLPFC, (**J**): Changes in FC strength between S1-DLPFC. * represents significant differences within groups between pre-test and post-test (* *p* < 0.05, ** *p* < 0.01). # denotes a significant difference between groups (#, *p* < 0.05).

According to the two-way ANOVA results in Fig. 4A, during the YBTs task with right leg support, there were no significant differences in the FC strength between the (PMC and SMC)-DLPFC brain regions among the three groups (*P* > 0.05). However, there was a significant difference in the FC strength between the pre-test and post-test (F_time_ = 8.745, *P* = 0.004), with the TCC group showing a significant increase in the FC strength between the (PMC and SMC)-DLPFC brain regions during the post-test (*P* < 0.05). In Fig. 4C, there were no significant differences in the FC strength between the (PMC and SMC)-M1 brain regions among the three groups (*P* > 0.05). However, there was a significant difference in the FC strength between the pre-test and post-test (F_time_ = 5.224, *P* = 0.023), with both the BW group and the TCC group showing a significant increase in the FC strength between the (PMC and SMC)-M1 brain regions during the post-test (*P* < 0.05). Figure 4G shows a significant difference in the FC strength between the FPA-S1 brain regions among the three groups (F_group_ = 6.987, *P* = 0.001). Bonferroni post-hoc comparisons revealed that the post-test FC strength between the FPA-S1 brain regions in the TCC group(0.002) was significantly higher than that in both the control group and the BW group (*P* < 0.05). Additionally, the interaction effect between group and time (F_group*time_ = 3.087, *P* = 0.048) was also significant (*P* < 0.05), with the TCC group showing a significant increase in the FC strength between the FPA-S1 brain regions during the post-test. However, there were no significant differences in the FC strength between the FPA-S1 brain regions in the three groups during the pre-test and post-test (*P* > 0.05). In Fig. 4H, the FC strength between the M1-S1 brain regions showed significant differences among the groups (F_group_ = 3.218, *P* = 0.042) and between the pre-test and post-test (F_time_ = 5.246, *P* = 0.023). Bonferroni post-hoc comparisons indicated that the post-test FC strength between the M1-S1 brain regions in the TCC group(0.036) was significantly higher than that in the control group, and the TCC group showed a significant increase in the FC strength between the M1-S1 brain regions (*P* < 0.05). In Fig. 4J, the FC strength between the S1-DLPFC brain regions showed significant differences among the groups (F_group_ = 5.231, *P* = 0.006) and between the pre-test and post-test (F_time_ = 6.776, *P* = 0.01). Additionally, the interaction effect between group and time (F_group*time_ = 4.064, *P* = 0.019) was also significant (*P* < 0.05). Bonferroni post-hoc comparisons revealed that the post-test FC strength between the S1-DLPFC brain regions in the TCC group(0.004) was significantly higher than that in the control group, and both the BW group and the TCC group showed a significant increase in the FC strength between the S1-DLPFC brain regions. In Fig. 4B, D, E and F, and 4I, the FC strength between the (PMC and SMC)-FPA, (PMC and SMC)-S1, FPA-DLPFC, FPA-M1, and M1-DLPFC brain regions showed no significant differences among the groups, nor were there significant differences between the pre-test and post-test comparisons (*P* > 0.05).

### Changes in laterality of YBTs, iEMG, and overall average brain FC strength between rois

**Fig. 5:**
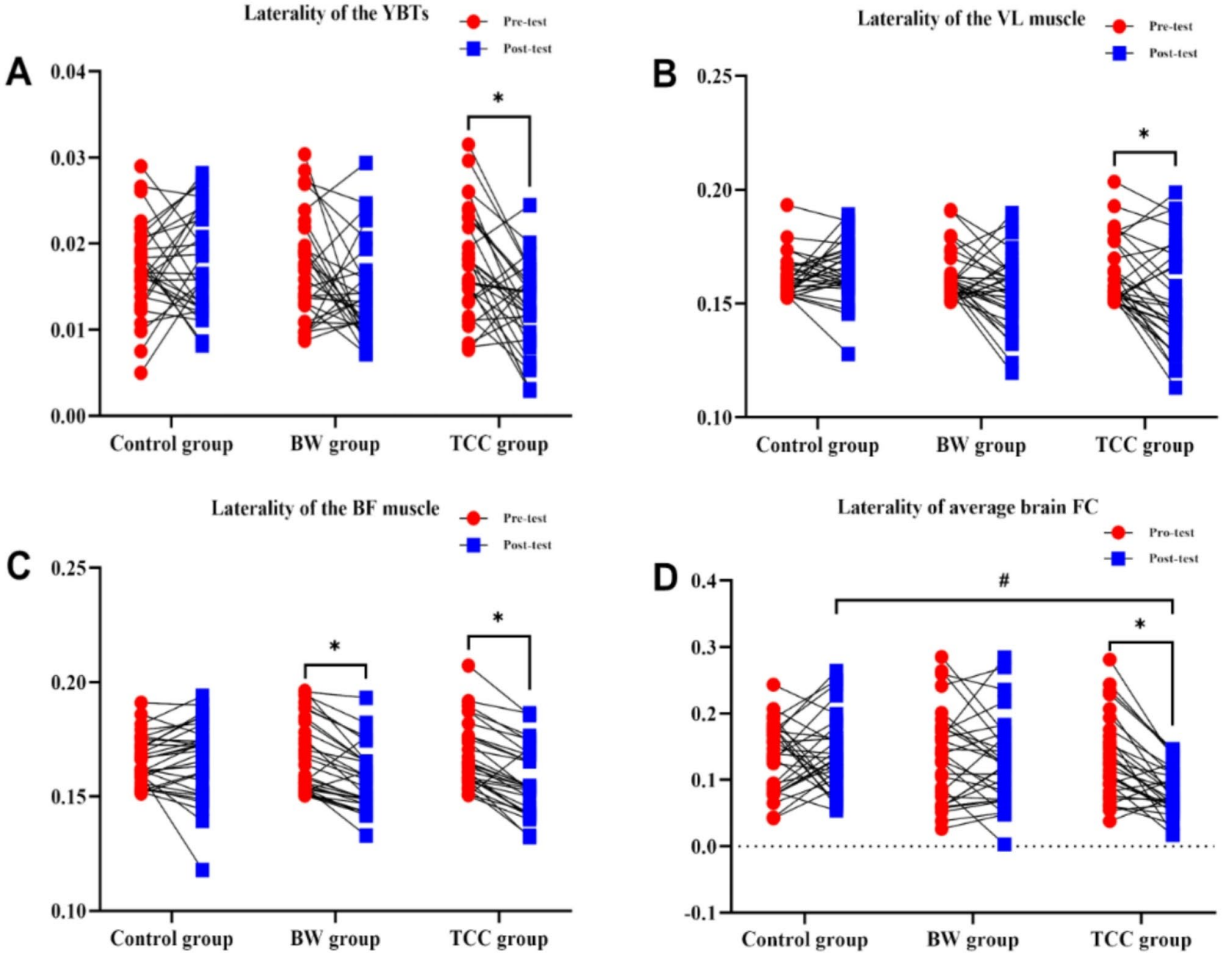
Changes in laterality of YBTs, iEMG, and overall average brain FC strength between ROIs. (**A**) shows the lateralization of the CS between the two-legged support during YBTs; (**B**) shows the lateralization of iEMG values for the VL muscle between the two legs; (**C**) shows the lateralization of iEMG values for the BF muscle between the two legs; (**D**) shows the lateralization of overall average brain FC strength during YBTs for both support legs. * represents significant differences within groups between pre-test and post-test (* *p* < 0.05, ** *p* < 0.01). # indicates a significant difference between groups (#, *P* < 0.05).

In Fig. 5A, the two-way ANOVA results for the laterality of the composite scores of the YBTs performed with bilateral support legs are described. There were no significant differences in the YBTs laterality values among the three groups (*p* > 0.05). However, there was a significant difference in the YBTs laterality values between the pre-test and post-test (F_time_ = 9.506, *P* = 0.002), and the interaction effect between group and time (F_group*time_ = 4.292, *P* = 0.015) was also significant. The TCC group showed a significant reduction in YBTs laterality values (*p* < 0.05). In Fig. 5B, the results of a two-way ANOVA for the laterality of the VL between the left and right legs during the YBTs. No significant differences were observed in the VL laterality values among the three groups (*p* > 0.05). However, significant differences were found in the VL laterality values between the pre-test and post-test (F_time_ = 9.569, *p* = 0.002), with the BW group and the TCC group showing a significant reduction in VL laterality values (*p* < 0.05). Figure 5C illustrates the results of a two-way ANOVA for the laterality of the BF between the left and right legs during the YBTs. No significant differences were observed in the BF laterality values among the three groups (*p* > 0.05). However, significant differences were found in the BF laterality values between the pre-test and post-test (F_time_ = 6.266, *p* = 0.013), and a significant interaction effect between group and time was also observed (F_group*time_ = 3.418, *p* = 0.035). Specifically, the TCC group demonstrated a significant reduction in BF laterality values (*p* < 0.05). In Fig. 5D, the two-way ANOVA results for the laterality of the average FC values during the YBTs performed with bilateral legs are described. There was a significant difference in the FC laterality among the three groups (F_group_ = 4.112, *P* = 0.018) (*P* < 0.05). Bonferroni post-hoc comparisons revealed that the post-test FC laterality in the TCC group was significantly lower than that in the control group(*P* = 0.022), with statistical significance. Additionally, there was a significant difference in the FC laterality between the pre-test and post-test (F_time_ = 4.0, *P* = 0.047), and the interaction effect between group and time (F_group*time_ = 3.345, *P* = 0.038) was also significant (*p* < 0.05). The TCC group showed a significant reduction in FC laterality.

## Discussion

This study investigated the effects of a 9-week TCC and BW training program on the EF, single-leg dynamic balance, the symmetry of bilateral lower limb muscle activation, the FC strength between regions of interest, and the symmetry of overall average brain FC in older adults. The results showed that: (1) The Stroop task results showed that only the TCC group exhibited a significant reduction in RTs, while the 2-back task results demonstrated that both the BW and TCC groups had significant reductions in RTs. Similarly, during the Stroop task, only the TCC group showed a significant increase in DLPFC activation, while during the 2-back task, the BW group exhibited a significant increase in HbO concentration in the DLPFC, and both the TCC group showed significant increases in HbO concentration in the DLPFC and FPA. (2) The BW group showed a significant improvement in single-leg dynamic balance ability in the left leg, while the TCC group showed significant improvements in single-leg dynamic balance ability in both the left and right legs. (3) During the right-leg YBTs, the FC strength between ROIs was significantly increased in both the BW and TCC groups, with some regions in the TCC group showing significantly higher FC strength compared to the BW and control groups. (4) During the left-leg YBTs, the FC strength between ROIs was significantly increased in both the BW and TCC groups, while some regions in the control group showed significantly decreased FC strength. (5) A laterality analysis was conducted by comparing the activation levels of the BF and VL (iEMG values) between the left and right legs, the YBTs scores between the left and right legs, and the overall average brain FC strength during the YBTs test for each leg. This study is the first to find that after TCC training, the laterality of single-leg dynamic balance ability, muscle activation, and overall average brain FC strength between the two legs was significantly reduced. In contrast, the BW group only showed a significant reduction in the laterality of the iEMG value of VL.

This study reveals that, compared to BW, TCC exhibits more significant effects in enhancing single-leg dynamic balance ability, increasing the neuromuscular activation level of lower limb muscles, and improving the CCI. The improvement in the symmetry of single-leg dynamic balance ability enhances the robustness of motor control, enabling older adults to promptly adjust their posture in response to changes in internal and external environments, thereby reducing the risk of falls^38^. With advancing age, older adults tend to adopt a co-contraction strategy during balance tasks to increase joint stiffness, thereby improving body stability and coordination^39,40^. This co-contraction pattern enhances the muscle spindles’ perception of proprioceptive information, allowing the brain to receive and process movement information more accurately^41,42^. Additionally, the increase in iEMG values may indicate improvements in muscle function and neuromuscular control in older adults. Therefore, changes in iEMG and CCI reflect the process by which older adults optimize postural control and enhance balance ability through multi-level regulation of the nervous and muscular systems. The significant functional improvements observed with TCC can be primarily attributed to its unique movement design. During practice, participants maintain a semi-squat posture and gradually shift their center of gravity from both legs to a single leg, while coordinating multi-directional body movements and upper limb actions. This series of movements requires precise perception of muscle contraction and tactile feedback, as well as the coordination of eccentric contractions (e.g., controlling the descent of the center of gravity) and concentric contractions (e.g., pushing against the ground when standing). This dynamic training approach effectively strengthens lower limb muscle strength, enhances muscle activation levels, and optimizes the coordination and stability between agonist and antagonist muscles^43^. In contrast, BW primarily involves flexion and extension movements of the ankle and knee joints, mostly occurring in the sagittal plane. This single-axis movement characteristic helps improve the function of the musculoskeletal system in the anterior-posterior direction but has limited effects on lateral balance^44^. Therefore, compared to BW, TCC’s precise joint control and muscle coordination contribute to better balance control.

The study demonstrates that both TCC and BW can effectively enhance working memory in older adults. As aerobic exercises, their mechanisms may involve an increase in plasma brain-derived neurotrophic factor (BDNF) levels, adaptive changes in the PFC at both structural and functional levels, and enhanced activation and synaptic plasticity in the PFC, collectively contributing to memory improvement^45,46^. However, in terms of inhibitory control, only TCC shows significant improvement. This difference may stem from the distinct effects of the two exercises on brain function and neural mechanisms. Compared to BW, TCC involves the learning and memorization of new skills and movement patterns, requiring practitioners to fully engage visual-spatial processing, motor memory, and inhibitory control abilities to execute complex motor coordination and task performance in dynamic environments^47^. Additionally, the meditative component of TCC enhances connectivity efficiency between the default mode network (DMN) and the DLPFC (primarily responsible for top-down executive control) during resting states, thereby improving self-regulation and executive control^48^. Meditation training can also induce higher theta rhythms in the frontal lobe, reduce cortisol levels associated with stress, and enhance immune function, helping practitioners improve emotional states, increase attention, and optimize autonomic nervous system activity^49,50^. In contrast, BW is a more automated form of exercise with lower demands on coordination and attention, relying less on higher central neural resources^51^. This may explain why TCC outperforms BW in improving EF. The enhancement of EF is closely related to the activation level of the PFC^52^. Consistent with the findings of this study, higher activation levels in the DLPFC and FPA are associated with better working memory, inhibitory control, and attention performance^53^. Therefore, regular practice of TCC not only significantly improves dynamic balance and reduces fall risk in older adults but also enhances EF and promotes optimization in related brain regions.

Postural control relies on the intricate processing and integration of sensory information at both spinal and supraspinal levels. With advancing age, the sensitivity, accuracy, and transmission speed of sensory systems such as vision, proprioception, and the vestibular system, which convey body position and balance signals to the central nervous system, gradually decline^54^. This degradation of sensory input may reduce the efficiency of automated postural control processing, thereby increasing the cognitive demand for maintaining posture^55^. When performing YBTs, older adults are required to reach the limits of single-leg dynamic balance stability, which prompts them to allocate more cognitive resources to maintain postural stability^56^ This process necessitates the coordinated involvement of bilateral cerebral cortices, ensuring both postural stability and the execution of complex motor planning, which is closely related to interhemispheric coordination and inhibitory mechanisms. Improvements in YBTs performance may reflect enhanced brain plasticity in older adults, indicating optimized neural function in motor control and balance regulation. Studies on BW and TCC training have revealed that effective connectivity between brain regions such as the PFC, S1, PMC, M1, SMC is significantly strengthened during the maintenance of single-leg dynamic balance^57,58^. Previous research has demonstrated that the FPA and DLPFC play critical roles in motor control, maintaining attention, integrating visual and proprioceptive information, and monitoring, planning, and adjusting movements^59–61^. The DLPFC is also involved in decision-making processes, helping to select optimal motor strategies. The SMC and PMC translate integrated sensory information into specific motor preparation or planning, ensuring the accuracy and timeliness of movements^62^. The M1, as the core region for motor execution, not only governs motor output but is also closely associated with the integration of sensory inputs such as proprioception and touch. Through precise muscle control and postural adjustments, it plays a vital role in maintaining and restoring balance^63^. The S1 is the primary region for processing sensory information, particularly proprioceptive and visual inputs. It receives sensory information from various parts of the body and transmits it to the M1, PMC, and SMC for motor control and postural adjustments^64^. The interactions among these brain regions form a complex neural network system, enabling the brain to effectively integrate sensory information, formulate motor strategies, and execute precise movement regulation in various situations, thereby facilitating rapid responses and maintaining postural stability.

This study found that the functional characteristics of this neural network system were further validated during the YBTs under both left and right leg support conditions. Specifically, only the TCC group exhibited a significant enhancement in the overall average symmetry of brain FC, indicating a notable improvement in brain coordination and synchronization among TCC practitioners when performing the same motor task with different lower limbs^65,66^. This phenomenon may suggest that, during unilateral lower limb support, the neural activation patterns in task-related brain regions of the TCC group demonstrate a high degree of inter-limb consistency. The mechanism underlying the reduced asymmetry in average brain FC strength in the TCC group may lie in the profound remodeling of higher-order neural networks through its multimodal training. TCC, through its symmetrical movements and meditation-guided cognitive-motor integration, may trigger Hebbian plasticity (co-activation strengthening connections), significantly increasing the FC strength of bilateral ROIs, as well as the GM volume and corpus callosum WM fiber density^19,67–69^. This may result in a high match between functional and anatomical connectivity, thereby reducing activation differences between the bilateral legs during YBTs tasks. In contrast, BW, which relies on the rhythmic alternating patterns of spinal central pattern generators (CPGs), lacks complex cognitive load and only induces limited GM growth and WM fine-tuning in the ROIs, leading to minimal improvement in brain functional symmetry^70^. The improved muscle activation symmetry observed in both groups may stem from the rhythmic regulation of spinal CPGs^71^. However, TCC further enhances this through slow and precise control, potentially more significantly activating monoamine neurotransmitter (e.g., serotonin/norepinephrine)-dependent persistent inward currents (PICs), synchronously recruiting bilateral high-threshold motor units, and combining “virtual-real transformation” dynamic loading to balance joint torque and muscle stiffness^72^. Additionally, compared to BW, TCC more prominently upregulates BDNF and IGF-1, delaying mitochondrial functional decline^73^. Therefore, compared to BW, the multimodal training of TCC demonstrates significant advantages in counteracting age-related compensatory decline. Moreover, TCC requires no special equipment or venue, and its movements are simple and easy to learn, making it suitable for promotion among older adults in both urban and rural areas. It can effectively improve their physical and mental health, enhance balance, and reduce social isolation, thereby enhancing their quality of daily life.

## Conclusion

This study, through a 9-week intervention experiment, demonstrated that TCC has significant advantages in improving dynamic balance, neuromuscular coordination, and brain FC symmetry in older adults. The results indicate that TCC training not only significantly enhanced single-leg dynamic balance but also reduced the laterality of bilateral lower limb muscle activation and optimized the symmetry of FC in cortical regions, thereby improving motor control and task performance. These improvements may be associated with the positive effects of TCC on GM and WM volumes in the brain, suggesting that it enhances neural adaptability and FC strength by promoting the formation of new neural network connections and activating brain regions related to motor control. In contrast, BW showed some effectiveness in improving working memory and partial muscle activation symmetry but was less effective than TCC in overall balance control and higher-order EF enhancement. Therefore, as a comprehensive exercise intervention, TCC can effectively enhance motor control, neural functional coordination, and quality of life in older adults, highlighting its significant health-promoting value.

### Limitations

This study did not account for the effects of exercise interventions on older adults of different age groups. Since different age groups may respond differently to exercise interventions, future research could explore the effects across various age ranges. Additionally, the methods used to measure brain FC might vary in sensitivity and specificity among individuals, potentially affecting the accuracy of the results. To enhance the precision of the research, future studies could integrate dynamic brain functional network data collected by fNIRS during active states with resting-state brain functional network data obtained from higher-resolution neuroimaging techniques such as functional magnetic resonance imaging (fMRI). This approach would enable a more in-depth analysis of the effects of exercise interventions on brain networks and the associated changes in neural plasticity. Therefore, future studies should consider longer intervention periods to comprehensively evaluate the long-term effects of exercise interventions.

## Electronic supplementary material

Below is the link to the electronic supplementary material.

Supplementary Material 1

Supplementary Material 2

Supplementary Material 3

Supplementary Material 4

Supplementary Material 5

## Data Availability

The data is provided within the supplementary information files.

## Abbreviations

A: Anterior
AS: Arterial stiffness
BF: Biceps femoris muscle
BW: BW
COP: Center of body weight
CR: Correction response
CS: Comprehensive score
DLPFC: Dorsolateral prefrontal cortex
FC: Functional connectivity
FM: Fat mass
fNIRS: Functional near-infrared spectroscopy
FPA: Frontal pole area
GM: Gray matter
CPGs: Central pattern generators
HbO: Oxygenated hemoglobin
HbT: Total hemoglobin
iEMG: Integrated myoelectric value
Lat: Lateralization
M1: Primary motor cortex
PL: Posterolateral
PICs: Persistent inward currents
PM: Posteromedial
PMC: Premotor cortex
PRE: Perceived rate of exertion
RH: Relative humidity
ROIs: Region of interests
ROS: Reactive oxygen species
RTs: Respond times
S1: Primary somatosensory cortex
SLM: Soft lean mass
SLS: Single leg stand
SMC: Supplementary motor cortex
TCC: TCC chuan
VL: Vastus lateralis muscle
YBTs: Y-balance tests
