# Supplementary material for: Effects of Tai Chi on executive function, single-leg dynamic balance, and brain functional connectivity in older adults
Source: Sci Rep. 2025 Apr 7;15:11838. doi: 10.1038/s41598-025-93321-w (PMC11976964; doi:10.1038/s41598-025-93321-w)

**Appendix A**

**Appendix A.1. Executive function test protocol**

(1)In the Stroop task, color names appear sequentially in the center of the computer screen, and the font colors are red, green, blue, or yellow. This experiment includes congruent conditions (e.g., the word “green” displayed in red font) and incongruent conditions (e.g., the word “green” displayed in green font). The four color words (red, green, blue, yellow) are randomly displayed on the screen, each in one of these four colors. Corresponding colored stickers for these words are placed on the Q, W, O, and P keys on the computer keyboard, allowing participants to select the correct answer.

(2) The 2-back task specifically tests the EF in elderly participants, focusing on working memory, response speed, and attention performance. In each task block, an uppercase English letter is shown in the center of a white screen. Participants need to judge whether the displayed letter matches the one shown two items prior, and respond quickly. If the letters match, they press the F key; if they do not, they press the J key. The test includes three blocks, each consisting of 18 trials, with a 30-second interval between blocks. The letter presentation time is 1.5 seconds, with a stimulus interval of 1 second, and the total test duration is 3 minutes and 15 seconds.

**Appendix A.2. The data analysis methods for fNIRS.**

fNIRS data were primarily preprocessed using the Nirspark system, developed by Danyang Huichuang Medical Equipment Co., Ltd. in China based on MATLAB software. The preprocessing steps were as follows: first, the raw NIRS light intensity was converted into optical density signals; motion artifacts were detected using the built-in function (with parameters set as tMotion = 1s; tMAsk = 2.0; STDEVthresh = 15.0; AMPthresh = 5.0); the detected motion artifacts were corrected using spline interpolation (hmrMotionCorrectSpline); a bandpass filter (0.01–0.1 Hz) was applied to remove physiological noise such as heartbeat and respiration, as well as baseline drift caused by environmental and temperature changes; the collected optical data were processed using the modified Beer-Lambert law to obtain filtered blood HbO signals. The Network module in the Nirspark software was used to calculate the blood HbO changes at various time points during the YBTs task, as well as the Pearson correlation coefficients of the blood HbO signal content in each region of interest over time. Finally, a Fisher-Z transformation was performed, and the transformed values were defined as the FC strength between channels and the overall average brain FC strength value.

**Appendix A.3. Y-balance tests protocol**

Participants must stand barefoot on one leg with their hands placed on the pelvis, stabilizing the right leg while extending the left leg as far as possible in three directions: anterior (A), posterior-medial (PM), and posterior-lateral (PL), and then return the leg to the starting position. Each direction is tested three times, with the maximum reach recorded for each trial. If any of the following situations occur, the test is considered a failure and needs to be retested: (1) the extended foot touches the ground, (2) the participant's center of gravity shifts to the extended leg, (3) the extended foot fails to return to the starting position, or (4) the hands are not maintained on the pelvis. Data from three successful trials are collected in each direction. The leg length is recorded by measuring the distance between the anterior superior iliac spine and the medial malleolus. The composite score is calculated by summing the maximum reach distances in the three directions and then dividing by three times the leg length on that side, using the formula:

composite score = [(A + PM + PL) / (Leg Length × 3)] × 100%.

**Appendix A.4. Calculation Steps for Normalized iEMG and CCI**

1. The iEMG was calculated using baseline normalization. First, participants performed maximum voluntary contractions (iEMG_MVC_) of the BF and VL in a quiet state, and the maximum values during the pre-test or post-test tasks (iEMG_task_) were recorded. The normalized values were then calculated using the normalization formula.


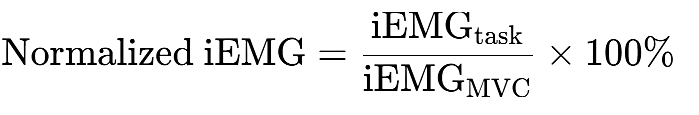


1. The co-contraction index (CCI) was calculated for the ipsilateral biceps femoris iEMG and vastus lateralis iEMG, using the following formula.

Calculation Steps

①Calculate the integrated EMG value of the antagonist muscle (Iant):


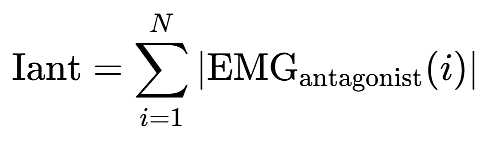


- EMG_antagonist_(*i*) is the EMG signal of the antagonist muscle at the *i*-th sample point.
- *N* is the number of sample points within the specific time period.

②Calculate the integrated EMG value of the agonist muscle (ITotal):


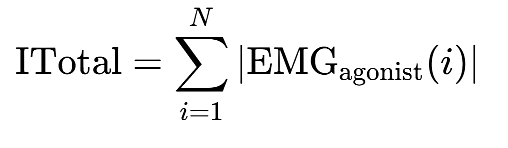


- EMG_agonist_(*i*) is the EMG signal of the agonist muscle at the *i*-th sample point.

③Calculate CCI

Substitute Iant and ITotal into the formula:


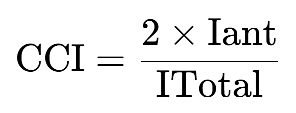

Supplement: Supplementary file 5 — Supplementary Material 5 [file 41598_2025_93321_MOESM5_ESM.docx]
